# Dynamic Time-Resolved Remodeling of the Immune Microenvironment After Resistance to BRAF/MEK Inhibitors in Melanoma: Mechanisms, Biomarkers, and Emerging Therapeutic Strategies

**DOI:** 10.3390/ijms27104484

**Published:** 2026-05-16

**Authors:** Wenjun Meng, Yan Liu, Haoran Zhang, Manting Wang, Xiaoli Mu, Ziqi Zhang, Yan Tie

**Affiliations:** 1 Department of Pain Management, West China Hospital, Sichuan University, Chengdu 610041, China; mwj1995@scu.edu.cn; 2 Outpatient Department, West China Hospital, Sichuan University, Chengdu 610041, China; 13547908816@163.com; 3 West China School of Medicine, Sichuan University, Chengdu 610041, China; 13157083691@163.com (H.Z.); v3185581067@163.com (M.W.); 4 Health Management Center, General Practice Medical Center, West China Hospital, Sichuan University, Chengdu 610041, China; mxli9509@163.com; 5 Department of Biotherapy, Cancer Center, West China Hospital, Sichuan University, Chengdu 610041, China

**Keywords:** melanoma, BRAF inhibitor, MEK inhibitor, acquired resistance, tumor immune microenvironment, immune escape

## Abstract

Targeted inhibition of the MAPK pathway with BRAF and MEK inhibitors (BRAFi/MEKi) produces rapid tumor regressions in BRAF V600-mutant melanoma, yet most patients ultimately develop acquired resistance. Resistance is not solely a tumor-cell-intrinsic phenomenon; it is accompanied by time-dependent remodeling of the tumor immune microenvironment (TIME) that can shape sensitivity to immune checkpoint inhibitors (ICIs) and inform rational combination or sequencing strategies. Early during MAPK inhibition, melanomas often display increased melanoma antigen expression and enhanced CD8^+^ T-cell infiltration, along with reduced immunosuppressive cytokines, suggesting a transient “immune-permissive” window. However, the same period can show induction of PD-L1 and T-cell exhaustion markers, foreshadowing adaptive immune resistance. At progression, immune-favorable features may diminish and immune evasion mechanisms, such as impaired antigen presentation and MHC-I downregulation, can become prominent and associate with resistance to immunotherapy. Here we review the temporal dynamics of TIME under MAPK inhibition, mechanistic links between resistance programs and immune remodeling, including signaling adaptation, focal adhesion/FAK signaling, dendritic cell dysfunction, antigen-presentation defects, and lymphatic/perilymphatic adipose remodeling, and practical biomarker opportunities across baseline, on-treatment, and progression timepoints. We also summarize emerging therapeutic strategies for post-resistance disease, including optimized ICI combinations, triple therapy concepts, and novel approaches such as combining FAK inhibition with RAF-MEK “clamp” therapy. Finally, we highlight key gaps and propose a framework for longitudinal sampling, standardized multi-omics integration, and TIME-informed trial design. The key distinguishing feature of this review is its time-resolved perspective on TIME remodeling, which links baseline immune contexture, early treatment-induced immune permissiveness, and the immune-evasive state that emerges during acquired resistance.

## 1. Introduction

Melanoma arises from melanocytes and is among the most aggressive and lethal forms of skin cancer, with its incidence closely linked to ultraviolet radiation exposure, genetic predisposition, and aberrant immune regulation [1]. Globally, its incidence continues to rise and is projected to increase significantly by 2040, while the prognosis for patients with advanced-stage melanoma remains extremely poor, with a 5-year survival rate of less than 10% [2]. The biological characteristics of melanoma include high metastatic potential, strong immunogenicity, and considerable molecular heterogeneity [3,4,5]. These features have made it one of the first solid tumors to be applied in immunotherapy and are frequently accompanied by mutations in BRAF, NRAS, c-KIT and other genes. For these reasons, melanoma has long been regarded as a model tumor for studies in tumor immunology and precision medicine [6].

BRAF V600 mutations define a major clinical subset of melanoma, and enable effective targeted therapy with BRAF/MEK inhibitors (BRAFi/MEKi) as the standard first-line treatment [7]. However, the vast majority of patients experience disease progression within 6–12 months of treatment initiation, and acquired resistance has become a bottleneck limiting its long-term efficacy [8,9,10]. Moreover, resistance mechanisms arising under BRAFi can confer cross-resistance to MEKi or combined BRAFi/MEKi, complicating salvage strategies and supporting the need for mechanism-based combinations [11,12]. In parallel, immune checkpoint inhibitors (ICIs) have transformed melanoma outcomes but responses are heterogeneous and resistance remains common, creating a clinical imperative to understand how targeted therapy reshapes antitumor immunity over time [13,14].

Accumulating evidence indicates that targeted therapy is not only cytotoxic to tumor cells but also exerts profound immunomodulatory effects on the tumor immune microenvironment (TIME) [15]. Early during BRAFi/MEKi treatment, MAPK pathway inhibition has been shown to enhance tumor antigen expression, increase CD8^+^ T-cell infiltration, and reduce immunosuppressive cytokines, thereby transiently promoting an immune-active state [16,17,18]. However, as resistance develops, melanoma cells undergo transcriptional and phenotypic reprogramming, which is frequently accompanied by T-cell exhaustion, loss of antigen presentation, and expansion of immunosuppressive cell populations, ultimately driving immune escape [18,19,20]. Accordingly, a dynamic model has emerged in which MAPK inhibition initially reconditions the TIME toward immune activation, whereas adaptive and acquired resistance re-establishes an immunosuppressive microenvironment [21,22].

Human paired-biopsy studies, mechanistic preclinical investigations, and integrative multi-omics analyses collectively support this paradigm, making it an opportune moment to systematically conceptualize “dynamic TIME remodeling following BRAFi/MEKi resistance” and translate it into actionable biomarkers and rational combination strategies. Accordingly, we organize the review around the temporal evolution of TIME rather than around resistance mechanisms alone. In this review, we also distinguish findings supported by clinical biopsies and patient cohorts from those derived from preclinical models and from computational analyses that are hypothesis-generating and require prospective validation.

## 2. Clinical and Biological Context: Resistance Is Heterogeneous and TIME-Relevant

The resistance mechanisms of melanoma to BRAFi/MEKi are highly heterogeneous, which directly leads to the diversity of clinical outcomes. For example, patients who develop resistance to first-line BRAFi/MEKi therapy show significant differences in their response to subsequent ICI therapy, with some patients still benefiting from it while others show cross-resistance [23]. This heterogeneity stems from multiple coexisting molecular mechanisms, including reactivation of the MAPK pathway (e.g., through NRAS, MEK1 mutations, or BRAF V600E amplification), activation of bypass signaling pathways (e.g., upregulation of EGFR, IGF1R/IR, and AKT/mTOR pathways), and epigenetic and metabolic reprogramming [24,25,26]. Resistant cells may also exhibit epithelial–mesenchymal transition (EMT) features and cancer stem cell-like characteristics, further enhancing their invasiveness and plasticity [27]. Therefore, understanding the heterogeneity of resistance mechanisms is key to developing effective subsequent treatment strategies.

Resistance to MAPK inhibition frequently involves reactivation of MAPK signaling through diverse genetic and non-genetic mechanisms, often heterogeneous within and between patients [11,28]. Even when BRAF and MEK are co-targeted, melanomas can acquire “combinatorial” resistance configurations (e.g., amplified mutant BRAF, MEK mutations) that restore pathway output and may yield distinct dependencies [29]. Such tumor-cell programs are not immunologically silent, because they alter antigen expression, cytokine/chemokine milieus, stromal interactions, and the abundance or function of immune subsets, thereby influencing both endogenous immunity and responsiveness to ICIs [30]. From a translational standpoint, the most informative question is not simply “What is the resistant genotype?” but “What is the resistant ecosystem?”—and how does it evolve from baseline to early on-treatment adaptation and finally to progression.

## 3. A Time-Resolved View of TIME Under MAPK Inhibition

### 3.1. Early On-Treatment “Immune-Permissive” Window

Human melanoma biopsies obtained shortly after initiating BRAFi (with or without MEKi) show increased melanoma antigen expression and increased CD8^+^ T-cell infiltrates, accompanied by decreased immunosuppressive cytokines such as IL-6 and IL-8 and increased markers of T-cell cytotoxicity [16,21,23]. These observations were derived from human melanoma biopsies and therefore represent clinical correlative evidence rather than proof of causality. These findings provide a mechanistic rationale for synergy between MAPK inhibition and immunotherapy, particularly if ICIs are deployed during this early window when T cells are present and tumor antigenicity is heightened [17].

In addition, BRAFi/MEKi can downregulate immunosuppressive cytokines secreted by tumor cells and reduce the recruitment of immunosuppressive cells such as myeloid-derived suppressor cells (MDSCs), further improving the immune status of TIME [21,31]. This early window may provide a rational period for combination or sequential immunotherapy before stable resistance is established, because the tumor’s visibility to the immune system is enhanced at this time, while acquired resistance mechanisms have not yet been fully established.

However, the same on-treatment samples can also show increased expression of exhaustion markers (e.g., PD-1 and TIM-3) and the immunosuppressive ligand PD-L1, implying that adaptive immune resistance can arise rapidly during MAPK inhibition [16]. Thus, the early immune-permissive state is best viewed as a mixed phenotype: enhanced antigenicity and infiltration coexisting with induced checkpoint pathways.

### 3.2. Early Adaptive Signaling and Transcriptional Remodeling

During continued MAPK inhibition, melanoma cells rapidly engage adaptive signaling and transcriptional programs that precede stable resistance. Central to this adaptive response is the activation of compensatory signaling pathways and metabolic rewiring [32]. Under the selective pressure of BRAFi/MEKi, tumor cells upregulate multiple receptor tyrosine kinases (RTKs), including EGFR, IGF1R/IR, and PDGFRβ, thereby restoring downstream MAPK or activating parallel pro-survival pathways such as PI3K-AKT-mTOR [24,25,26]. Concurrently, transcription factors such as STAT3 are frequently activated, forming extensive crosstalk with MAPK signaling and reinforcing resistance-associated phenotypes [33].

Beyond these steady-state adaptations, recent high-resolution temporal analyses have revealed that early responses to oncogenic BRAF inhibition occur in a phased manner, with rapid transitions toward a quiescent-like state accompanied by the compensatory activation of Src family kinase signaling as a dominant adaptive module [34]. This dynamic rewiring not only sustains tumor cell survival but also reshapes cellular stress responses and secretory programs. At the metabolic level, drug-tolerant persister cells undergo a shift from glycolysis toward oxidative phosphorylation, with increased reliance on fatty acid β-oxidation, including peroxisomal pathways, to support energy demands and survival [35].

Collectively, these early, non-genetic adaptive processes provide a survival advantage and establish a permissive foundation for subsequent stable resistance. Importantly, such adaptive signaling and metabolic reprogramming are likely to influence tumor-immune interactions by modulating cytokine secretion, antigen presentation, and cell-surface signaling, thereby linking early resistance evolution to subsequent remodeling of the TIME.

### 3.3. Progression/Resistance State: Loss of Immune-Favorable Features and Emergence of Immune Escape

During the progression of resistance after BRAFi monotherapy, studies have observed a significant decrease in melanoma antigen expression levels and CD8^+^ T cell infiltration; in some cases, this immunosuppressive phenotype can be reversed by combining BRAFi/MEKi, suggesting that TIME has significant dynamics and is treatment-dependent [16]. Furthermore, immune escape gradually intensifies during disease progression, and one of its mechanisms is impaired antigen presentation. Pathological studies have shown that downregulation of major histocompatibility complex class I molecules (MHC-I) is prevalent in advanced melanoma and is closely related to reduced CD8^+^ T cell infiltration, tumor metastasis, and resistance to ICI therapy [36]. Therefore, in the context of MAPK inhibitor (MAPKi) resistance, MHC-I and antigen presentation pathways are not only important biomarkers but also constitute a mechanistic hub connecting targeted therapy failure and immunotherapy failure.

As the disease progresses to complete drug resistance, TIME undergoes profound and stable remodeling, characterized by the closure of the early “immune-permissive” window and the synergistic activation of multi-level immune escape mechanisms [37]. On the one hand, the MAPK pathway is usually reactivated through mechanisms such as BRAF amplification, NRAS/KRAS mutation or MEK mutation, driving continuous tumor proliferation; on the other hand, the tumor microenvironment (TME) gradually shifts to a highly immunosuppressive state [16,21,23]. Specifically, tumor cells systematically suppress anti-tumor immune responses by upregulating immune checkpoint molecules (such as PD-L1), secreting immunosuppressive cytokines (such as IL-8, IL-1β), and recruiting immunosuppressive cell groups such as regulatory T cells (Tregs) and tumor-associated macrophages (TAMs, especially M2 type). For example, activation of transcription factor GLI1 can promote the recruitment and expansion of polymorphonuclear MDSCs and reduce dendritic cell (DC) and effector T cell infiltration, further strengthening the immunosuppressive microenvironment [24]. Furthermore, activation of the EMT not only enhances tumor invasion and metastasis but also promotes immune escape and exacerbates resistance to targeted and immunotherapies by reshaping tumor-immune cell interactions [38]. Ultimately, the solidification of this immunosuppressive TIME leads to significant cross-resistance to subsequent ICI therapy [23].

## 4. Mechanisms Linking MAPKi Resistance to TIME Remodeling

### 4.1. Tumor-Intrinsic Resistance Programs That Shape Immunogenicity

Resistance frequently restores MAPK output or activates bypass programs, changing differentiation state, antigen expression, and cytokine production. Classic resistance mechanisms to BRAFi include NRAS mutations, BRAF alterations, EGFR-STAT signalings, IGF1R/IR, and other pathway reactivation routes, often coexisting within resistant tumors [10,11,23,28]. Under BRAFi/MEKi pressure, resistant configurations can be “tunable” and combinatorial (e.g., ultra-amplified mutant BRAF with CRAF activation plus MEK mutants), emphasizing that resistant tumor cells can occupy multiple stable states that plausibly correspond to distinct immune ecologies [29].

### 4.2. Antigen Presentation and MHC-I Downregulation as Immune-Escape Axes

The effective anti-tumor activity of CD8^+^ T cells relies on antigen presentation mediated by MHC-I molecules; however, defects in the antigen presentation machinery, particularly the downregulation of MHC-I, serve as a pivotal axis enabling immune evasion in MAPKi-resistant melanoma [39]. Studies indicate that reduced MHC-I expression is not only closely associated with diminished CD8^+^ T-cell infiltration but also significantly correlated with resistance to ICI therapy [36]. When tumors acquire resistance under the selective pressure of targeted therapy, this process is often accompanied by the remodeling of intrinsic cellular programs (such as MAPKi-induced cellular state transitions and the activation of dedifferentiation trajectories). This process can lead to the loss of differentiation antigens and further impair MHC-I-mediated antigen presentation capabilities. Consequently, resistant tumors evade immune surveillance by reducing their “antigen visibility” on the one hand, while simultaneously exhibiting reduced infiltration of effector T cells on the other; this establishes a “double barrier” characterized by both recognition defects and insufficient infiltration, which collectively limits the efficacy of monotherapies targeting PD-1/PD-L1.

Thus, MHC-I downregulation is not merely a consequence of immune editing; rather, it serves as a critical bridge linking targeted therapy resistance with immunotherapy resistance, thereby highlighting the significant potential of combination therapeutic strategies aimed at restoring antigen presentation or introducing alternative immune effector mechanisms [40].

### 4.3. Dendritic Cell (DC) Function Under MAPK Pathway Modulation

The MAPK pathway exerts a regulatory influence on the function of DCs, thereby serving as a critical nexus linking targeted therapy with anti-tumor immune responses. As professional antigen-presenting cells, the recruitment, maturation, and expression of co-stimulatory molecules by DCs directly determine the efficiency of T-cell priming. Preclinical studies have demonstrated that BRAFi/MEKi can remodel the TME and exert immunomodulatory effects [41].

Under certain conditions, they are capable of reversing tumor-mediated suppression of DC function: for instance, in BRAF V600E melanoma models, blockade of the MAPK pathway restores DC cytokine secretion and co-stimulatory molecule expression, thereby enhancing the capacity for T-cell priming [42]. However, this immune-enhancing effect is markedly “context-dependent”: in the setting of resistance to MAPKi, the TME often shifts toward an immunosuppressive state. For example, the activation of GLI1 within tumor cells can suppress DC infiltration and activation by modulating the secretion of chemokines (such as CCL7), whereas the silencing of GLI1 can promote the activation and cytoskeletal remodeling capabilities of monocyte-derived DCs [24]. Furthermore, different MAPKi exhibit varying direct effects on DCs; for instance, MEKi may compromise DC survival and T-cell priming functions, whereas BRAFi (such as vemurafenib) demonstrate comparatively lower intrinsic toxicity toward DCs [42].

Overall, MAPK-pathway effects on DCs are context dependent and vary with the target node, dose, timing, and cellular exposure. This inherent complexity holds instructive value for optimizing combination strategies involving targeted therapy and immunotherapy.

### 4.4. Cytokines, Myeloid Remodeling, and Checkpoint Induction

In the early stages of MAPKi therapy, the TME can undergo remodeling that is conducive to immune activation, such as a decrease in IL-6 and IL-8 levels and an increase in CD8^+^ T cell infiltration, supporting the model that MAPKi can reduce immunosuppressive inflammatory signals and improve anti-tumor immune status [16]. Preclinical studies have also shown that combining MAPKi with immunotherapy can further enhance T cell infiltration and anti-tumor activity, thus providing a theoretical basis for time-window-based combination strategies [17].

However, this immune activation effect is often accompanied by the emergence of adaptive immune regulation: PD-L1 and T cell fatigue-related markers are gradually upregulated during treatment, suggesting that immune checkpoint induction is an important feedback mechanism and also constitutes a basis for the early introduction of ICIs [16]. With drug resistance developing, tumors further actively shape an immunosuppressive microenvironment by secreting specific cytokines and chemokines. For example, Bcl-xL overexpression can upregulate IL-8 and IL-1β through the NF-κB-dependent pathway, inducing macrophages to polarize towards the M2 phenotype and promoting their recruitment [43]. Meanwhile, midline protein (MDK) can reprogram the microenvironment into an inflammatory but immune-tolerant state by coordinating NF-κB activation and interferon pathway inhibition, driving macrophages to transform into a tolerant phenotype and promoting CD8^+^ T cell dysfunction [25]. In addition, the immune checkpoint profile is also remodeled during the drug resistance stage. For example, the increased proportion of TIM-3^+^ T cells indicates that alternative immunosuppressive pathways other than PD-1 are activated [26].

In summary, in the dynamic evolution from early immune activation to immunosuppression during the drug resistance stage, cytokine-driven myeloid cell remodeling and adaptive induction of checkpoint molecules together constitute the potential mechanism connecting targeted therapy resistance and systemic immunosuppression.

### 4.5. Focal Adhesion/FAK Signaling as an Interface Between Resistance and Immune Evasion

The focal adhesion/focal adhesion kinase (FAK) signaling pathway, as an important regulator of extracellular matrix (ECM) sensing and cell migration, constitutes an important interface connecting MAPKi resistance and immune escape [44]. Previous studies have shown that FAK is one of the key regulators of EMT [38]. Based on this, the latest mechanistic studies have provided further direct evidence: transcriptome analysis of BRAFi/MEKi-resistant melanoma showed that focal adhesion signaling was significantly activated, and MAPKi (including RAF-MEK with avatotinib) can adaptively activate FAK through the MAPK-RhoE feedback mechanism, thereby driving the continuous activation of the RhoA-FAK-AKT signaling pathway and forming a convergent survival pathway that promotes tumor cell survival [45]. Functionally, this axis not only enhances the anti-apoptotic ability and migration potential of tumor cells (drug resistance phenotype), but may also establish physical and functional immune rejection barriers (immune escape phenotype) by altering tumor–matrix interactions. Importantly, the combined use of FAK inhibitors and avetotinib can significantly enhance the pro-apoptotic effect and overcome drug resistance in BRAFi/MEKi resistance and immunotherapy resistance models [45].

**Figure 1:**
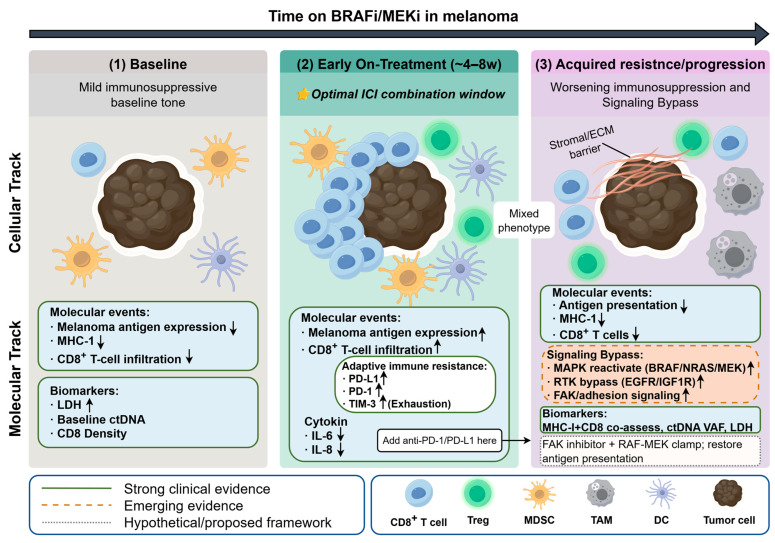
Schematic illustration of the time-dependent remodeling of the tumor immune microenvironment (TIME) during BRAF/MEK inhibitor (BRAFi/MEKi) treatment in melanoma. In the pretreatment state, melanoma lesions are characterized by high tumor burden, limited CD8^+^ T-cell infiltration, low antigen presentation, and a relatively immunosuppressive microenvironment. During early on-treatment exposure, MAPK pathway inhibition induces a transient immune-permissive window, marked by increased melanoma antigen expression, enhanced CD8^+^ T-cell infiltration, reduced immunosuppressive cytokines such as IL-6 and IL-8, and improved dendritic cell-mediated immune activation. However, adaptive immune resistance can emerge rapidly, accompanied by upregulation of PD-L1 and exhaustion-associated markers such as PD-1 and TIM-3. With acquired resistance and disease progression, the immune-favorable state is progressively lost, leading to diminished antigen presentation, downregulation of MHC-I, reduced effector T-cell infiltration, and expansion of immunosuppressive populations including regulatory T cells (Tregs), myeloid-derived suppressor cells (MDSCs), and tumor-associated macrophages (TAMs). This dynamic model highlights the temporal window in which combined or sequential immunotherapy may be most effective.

Overall, these findings support a mechanistic hypothesis that FAK signaling is not only a promising molecular hub connecting targeted therapy resistance and immunosuppression, but also represents a potential dual intervention target that can simultaneously reshape the TIME and enhance immunotherapy sensitivity. Figure 1 shows the dynamic remodeling of the TIME during BRAFi/MEKi therapy and resistance in melanoma.

### 4.6. Lymphatic Remodeling and Perilymphatic Adipose Tissue as a Spatial Compartment of TIME

Beyond T-cell and myeloid programs, the lymphatic compartment represents a spatially distinct and biologically important layer of the melanoma immune microenvironment [46,47]. In a landmark study, lymphatic vessels were shown to regulate immune microenvironments in both human and murine melanoma, with lymphatic remodeling influencing cytokine expression, leukocyte infiltration, and the efficacy of cytotoxic T-cell responses, indicating that lymphatics are not merely metastatic conduits but active regulators of local immunity [48]. Fankhauser et al. also stated that tumor lymphangiogenesis can promote T cell infiltration and enhance the immunotherapy response [49]. This is particularly relevant in BRAFi/MEKi-resistant disease, where altered antigen drainage, impaired immune-cell trafficking, and changes in tumor-draining lymph node signaling may further constrain effective immune priming.

The adjacent adipose compartment should also be considered. The lymphatic-adipose axis is increasingly recognized as a reciprocal regulatory niche: defective lymphatic function can promote adipose inflammation, fibrosis, and immune dysregulation, whereas adipose-derived stromal cells can support lymphangiogenic programs and lymphatic endothelial cell behavior [50,51,52,53]. Together, these findings suggest that perilymphatic adipose tissue may contribute to a suppressive melanoma niche and should not be overlooked in spatially resolved analyses of TIME. More recent experimental data further show that disruption of the tumor-draining lymphatic network can reduce local tumor control and effector-like T-cell infiltration, reinforcing the importance of functional lymphatic transport for antitumor immunity [54]. Accordingly, lymphatic remodeling should be considered alongside T-cell and myeloid remodeling as an additional spatial dimension of melanoma TIME.

### 4.7. Type 2 Immunity as an Emerging Non-Classical Immune Pathway in Melanoma TIME

In addition to canonical type 1/T-cell-centered and myeloid-centered circuits, type 2 immunity may represent an additional layer of melanoma immune regulation [55]. Although type 2 responses have historically been viewed as context-dependent or even pro-tumor in some settings, recent works suggest that they can also support antitumor immunity and influence therapeutic responsiveness [56]. In melanoma models, IL-4 was shown to modulate type 1 interferon signaling and restore T cell-mediated tumor rejection, indicating that type 2-associated programs can recondition the TIME rather than merely suppress it [57]. Group 2 innate lymphoid cell (ILC2) infiltration has been associated with favorable outcomes, and experimental activation of the ILC2-eosinophil axis can enhance anti-tumor responses, including in the setting of PD-1 blockade [55,58,59]. More broadly, recent reviews argue that orchestrating type 1 and type 2 immunity may provide a more effective framework for cancer immunotherapy than type 1-centric approaches alone [56]. Accordingly, type 2 immunity should be considered an emerging modulatory axis in melanoma immunotherapy, although prospective clinical validation remains limited.

## 5. Biomarkers for Post-Resistance TIME States: What to Measure, When to Measure

### 5.1. Principles: Longitudinal, Multi-Compartment, and Actionable

Biomarkers for post-resistance TIME should integrate longitudinal, spatial, and clinically actionable information. First, longitudinal dynamic monitoring is crucial because TIME continues to evolve during MAPKi, with significant differences at different stages; for example, rechallenge studies have shown that treatment intervals are associated with survival, suggesting that TIME may remodel during drug withdrawal [9].

Second, spatial heterogeneity needs to be considered, as different metastatic sites (such as liver, brain, lung, etc.) have different immune infiltration characteristics, which affect prognosis and response to ICIs [26]. Liver and brain metastases are typically less immunogenic than lung or lymph node metastases, with lower T-cell infiltration and greater spatial separation between immune cells and melanoma cells [26]. In treatment-naïve melanoma, liver metastases showed the lowest proportion of PD-1^+^ T cells and higher TIM-3 expression, whereas brain metastases displayed the highest proportion of PD-1^+^ T cells but the lowest macrophage density [26,60]. These differences suggest that biomarkers such as PD-L1, TIM-3, and TIL density may not have uniform meaning across metastatic sites, and that site distribution should be incorporated alongside circulating markers such as LDH, S100, and NLR in biomarker models [60,61].

Third, biomarkers should be operable, such as ctDNA clearance, which can serve as an important indicator for early prediction of efficacy and guidance of treatment adjustment [62,63].

Furthermore, the ideal strategy should cover both tumor and immune components and support dynamic assessment at multiple time points to guide key clinical decisions (such as combined ICI or strategy switching); at the same time, developing a detection platform that can quantify receptor-ligand interactions and retain spatial information is particularly crucial for improving the predictive ability of ICI [14].

### 5.2. Tissue-Based Biomarkers

Practical tissue biomarkers supported by current evidence include: CD8^+^ T-cell infiltration increases early on treatment while decreases can accompany progression [16,64]; PD-L1 and exhaustion markers (PD-1, TIM-3) induce on treatment, supporting early checkpoint blockade concepts [16]; MHC-I expression downregulation associates with decreased CD8 infiltration, metastatic spread, and ICI resistance, making it a candidate marker in resistant tumors [36]. Importantly, the interpretation of tissue biomarkers should be contextualized by metastatic site, because liver and brain metastases can show distinct immune-cell densities, checkpoint expression patterns, and spatial relationships to melanoma cells.

In addition, specific gene expression signatures have predictive value. For example, a panel of 13 gene signatures, including HMOX1, ICAM, MMP2, and SPARC, can identify melanoma cases that are resistant to or have progressed on BRAFi/MEKi with more than 70% accuracy [65]. Another example is TERT expression, which is associated with resistance to MAPKi and may become a new biomarker of resistance and a therapeutic target [66].

### 5.3. Multi-Omic and Integrative Biomarkers: The Value of Harmonized Datasets

Combined proteomic and transcriptomic analysis of drug-resistant cell lines not only identified drug-resistant secretory proteins (such as plasminogen activator inhibitor-1 and thymosin β-4), but also revealed that reprogramming of metabolic pathways such as oxidative phosphorylation and cell cycle is an important drug resistance mechanism [67]. In addition, bioinformatics mining using public databases can construct feature models related to the TIME and prognosis. For example, to construct a prognostic feature, immune-related long non-coding RNAs (ireRNAs) are associated with the survival time and unique TME of melanoma patients [68].

Multi-cohort resources can accelerate the discovery of biomarkers for MAPKi response and resistance. MelanoDB aggregates nine high-grade melanoma cohorts (*n* = 417) receiving MAPKi therapy, including pre-treatment and progression sampling, along with sequencing and gene expression subsets, and provides web applications for exploration [8]. Such coordinated datasets are particularly important for modeling baseline predictors of response duration and for comparing pre- and post-treatment molecular signatures to infer acquired resistance mechanisms. Importantly, MelanoDB also highlights the need to correct for batch effects in cross-study gene expression analyses, a requirement for reproducible biomarker development. Multi-omic and bioinformatic analyses can generate testable hypotheses for TIME remodeling, but prospective validation in clinical samples is still needed.

### 5.4. Adjunct/Clinical Biomarkers

Non-invasive or clinically proximal biomarkers are suitable for repeated monitoring. For example, dermoscopic regression in melanocytic lesions has been reported to be associated with ICI response in metastatic melanoma, suggesting that readily detectable phenotypes may reflect underlying immune activity, although such signals require careful validation and may be context-dependent [69]. The dynamic changes in variant allele frequencies (VAFs) of ctDNA can reflect tumor burden and response to treatment. BRAF VAF and its tumor cell proportion-corrected normalized VAF (nVAF) are independent predictors of patients’ survival with metastatic melanoma treated with BRAFi/MEKi [70]. In addition, traditional serum biomarkers such as lactate dehydrogenase (LDH) still have prognostic significance, with high levels of LDH associated with poorer response to BRAFi/MEKi rechallenge [9]. Treatment-related adverse events (TRAEs) can also serve as clinical biomarkers. For example, immune-related adverse events such as vitiligo and thyroiditis during ICIs treatment, or fever and skin toxicity during BRAFi/MEKi treatment, are often associated with better treatment response [62].

To facilitate clinical interpretation, Table 1 summarizes the major resistance mechanisms, their effects on the TIME, candidate biomarkers, evidence levels, and therapeutic implications across the treatment timeline.

## 6. Therapeutic Strategies After BRAFi/MEKi Resistance: TIME-Informed Opportunities

### 6.1. Checkpoint Inhibitor Combinations and Timing

The early immune-permissive window under MAPK inhibition (antigen upregulation and increased CD8 infiltration) provides a strong rationale for adding ICIs during targeted therapy rather than waiting for progression [16,23]. Preclinical study shows that combining BRAFi and MEKi with immunotherapy can improve antitumor activity, increase T-cell infiltration, and yield superior efficacy when anti-PD-1 is incorporated in triple therapy contexts [17].

Nevertheless, toxicity has historically limited some combinations, and the schedule (concurrent vs. staggered) remains a design variable. The sequence of immunotherapy followed by BRAFi/MEKi (encorafenib/binimetinib) significantly prolonged progression-free survival of new brain metastases compared to the reverse sequence; placing immunotherapy in a “sandwich” manner between BRAFi/MEKi therapy (i.e., BRAFi/MEKi for 8 weeks, followed by ICI, and then BRAFi/MEKi back after disease progression) also showed a protective effect against brain metastases [71]. This evidence highlights the importance of optimizing the sequence of targeted and immunotherapy based on TIME dynamics to maximize clinical benefit.

Metastatic-site heterogeneity should also inform therapeutic selection and sequencing. Brain metastases raise additional considerations related to blood–brain barrier (BBB) penetration, corticosteroid exposure, and local control with surgery or stereotactic radiosurgery, whereas liver metastases are often associated with a more immunosuppressive microenvironment and reduced responses to PD-1-based therapy [72,73,74]. Accordingly, patients with liver or brain metastases should not be pooled with other metastatic patterns when evaluating response to combination therapy, and metastatic site should be included as a stratification factor in future biomarker-driven trials [26]. Table 2 summarizes the metastatic site heterogeneity and its biomarker and therapeutic implications in melanoma.

### 6.2. Targeting Adaptive Signaling and Tumor–Matrix Survival Programs

Mechanistic studies of early adaptation nominate signaling nodes (e.g., Src family kinase induction) as combinable vulnerabilities during the adaptation phase [34]. In resistant disease, focal adhesion/FAK activation appears to be a recurrent survival program and can be therapeutically exploited. The combination of FAK inhibition with the RAF-MEK clamp avutometinib overcame resistance to targeted and immune therapies in multiple melanoma models, supporting clinical development of such combinations in post-resistance settings [45].

Overall, Table 3 summarized the time-resolved TIME remodeling under BRAFi/MEKi at baseline, early on-treatment, and progression.

### 6.3. Addressing Antigen Presentation Defects

**Figure 2:**
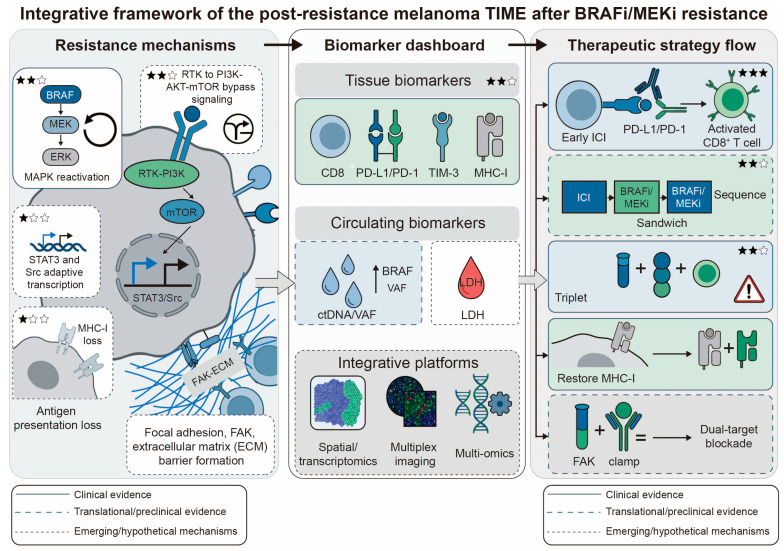
Integrative framework linking resistance mechanisms, biomarker development, and therapeutic strategies in melanoma after BRAFi/MEKi resistance. The left panel summarizes major tumor-intrinsic and microenvironmental resistance programs, including MAPK pathway reactivation (e.g., NRAS, MEK, or BRAF alterations), bypass signaling through RTKs and PI3K-AKT-mTOR, transcriptional adaptation (e.g., STAT3 and Src family kinase signaling), loss of antigen presentation, and focal adhesion/FAK-mediated survival signaling. The middle panel highlights biomarker categories that may guide patient stratification and real-time monitoring, including tissue-based markers (CD8, PD-L1, PD-1, TIM-3, MHC-I), circulating biomarkers (ctDNA, BRAF variant allele frequency, LDH), and integrative multi-omic or spatial platforms. The right panel illustrates TIME-informed therapeutic strategies, such as early immune checkpoint inhibitor (ICI) combination, optimized sequencing of targeted therapy and immunotherapy, triplet regimens, restoration of antigen presentation, and novel combinations targeting adaptive survival pathways, including FAK inhibition plus RAF-MEK clamp therapy. Together, these interconnected nodes emphasize that resistance should be viewed not only as a tumor-cell-intrinsic event but also as an ecosystem-level process that can be exploited for biomarker-guided precision therapy.

Given the association of MHC-I downregulation with reduced CD8 infiltration and ICI resistance, strategies that restore antigen presentation or recruit immune effector mechanisms less dependent on classical MHC-I presentation may be required in a subset of resistant tumors [36]. In practice, measuring MHC-I and CD8 jointly may help triage patients toward therapies aiming to reinflate T-cell infiltration and/or restore tumor visibility. Collectively, Figure 2 shows the mechanisms, biomarkers, and emerging therapeutic strategies in post-resistance melanoma.

### 6.4. Sequencing, Re-Challenge, and Cross-Resistance Considerations

Clinical data indicate that MEKi monotherapy has minimal activity after prior BRAFi exposure, consistent with shared resistance mechanisms and supporting the concept of cross-resistance within the MAPK axis [12]. Experimental systems also show that resistance mechanisms to BRAFi can confer resistance to combined BRAFi/MEKi, reinforcing that simply “stacking” pathway inhibitors may not overcome resistance without targeting the emergent dependencies (e.g., FAK/AKT signaling, tumor–matrix interactions, or immune escape routes) [11,45].

### 6.5. Clinical Caveats and Unresolved Translational Issues

Although triple therapy, sequencing, rechallenge, FAK inhibition, and RAF-MEK clamping are biologically attractive strategies, their clinical translation remains constrained by several limitations. First, combination approaches may be associated with substantial toxicity and treatment discontinuation. For example, in a real-world melanoma cohort treated with second-adjuvant BRAF/MEK inhibition after adjuvant PD-1 therapy, 72% of patients required dose adjustments, 23% experienced grade ≥ 3 toxicity, and 38% permanently discontinued treatment because of toxicity [75].

Second, treatment benefit appears to vary across patient subgroups and metastatic sites: symptomatic brain metastases and elevated LDH were associated with less benefit from BRAFi/MEKi rechallenge, while longer treatment-free intervals were associated with improved outcomes; similarly, mucosal melanoma was associated with shorter time to progression after ICI discontinuation [9,76].

In addition, the available evidence for triple therapy and related combinations remains heterogeneous, and severe toxicities, including grade 3–5 immune-related adverse events, have been reported in real-world cohorts [77,78].

Finally, proposed biomarkers are largely exploratory and lack prospective validation; recent transcriptomic and genomic prediction models are promising but still require external confirmation in multicenter prospective studies [79,80,81]. Therefore, the optimal timing and sequencing of these interventions remain unresolved and should be addressed in biomarker-driven prospective trials [82].

## 7. Key Gaps and Future Directions

### 7.1. Standardized Longitudinal Sampling

Existing studies largely rely on single-timepoint biopsies, failing to capture the continuous evolution of immune cell composition and functional status during drug resistance. For example, a longitudinal analysis of patients with acral melanoma brain metastases revealed dynamic changes in the TME during immunotherapy and targeted therapy resistance, highlighting the importance of continuous sampling for elucidating drug resistance mechanisms [83]. The field needs more paired and triplet biopsies (baseline/on-treatment/progression) with matched blood and imaging, enabling TIME trajectory mapping rather than static correlates [16].

### 7.2. Spatially Resolved Biomarkers

The degree of T cell infiltration in melanoma liver and brain metastases is significantly lower than that in lung or lymph node metastases, and the spatial distance between T cells and tumor cells is greater, which may be one of the reasons why these sites respond poorly to immunotherapy [26]. Predictive biomarker frameworks increasingly emphasize spatial measurements of immune-tumor interactions and receptor-ligand dynamics [14]. Therefore, future research should widely apply and develop technologies such as multiplex immunofluorescence, imaging mass cytometry (CyTOF-IMC), and spatial transcriptomics [84].

### 7.3. Integrative Multi-Omic Modeling with Batch-Aware Methods

Batch effects are a challenge limiting the reliability and reproducibility of results when integrating multi-omics data from different platforms and laboratories. Therefore, developing and applying batch-aware methods is crucial for robustly identifying resistance drivers and biomarkers from large-scale, multicenter cohorts, and providing a computational biology foundation for mechanistic stratification of therapy. Integrative resources like MelanoDB not only support larger-scale data analysis but also further highlight the technical challenges that must be addressed in the development of clinical-grade biomarkers, including batch effects, platform heterogeneity, and uneven sampling time points [8].

### 7.4. Mechanism-Stratified Trials

Given heterogeneous resistance programs (MAPK reactivation, focal adhesion/FAK activation, antigen-presentation loss), future trials should incorporate biomarker-driven stratification and adaptive designs rather than one-size-fits-all combinations [29,45]. For example, EGFR inhibitors combined with immunotherapy may be a promising strategy for drug-resistant patients with activated EGFR signaling pathways [23]. Similarly, clinical trials targeting specific resistance mechanisms, such as IGF1R/IR signaling [10], TERT overexpression [66], or specific gene signatures [65], are urgently needed. Such mechanism-stratified trial designs are expected to improve the objective response rate of subsequent treatments and match the most effective individualized treatment regimens for patients in different drug-resistant subgroups.

## 8. Conclusions

Melanoma TIME should be viewed as a spatially and temporally dynamic ecosystem that includes tumor-intrinsic, immune, lymphatic, nodal, and adipose compartments. The field now needs to move beyond descriptive association toward prospective biomarker validation in longitudinally sampled patient cohorts. In particular, serial multi-compartment profiling should be used to determine whether ctDNA kinetics, MHC-I/CD8 status, PD-L1/TIM-3 expression, and metastatic-site-specific immune features can guide treatment timing, sequencing, and rechallenge decisions. Future clinical development should also prioritize mechanism-stratified trials for resistance states driven by MAPK reactivation, antigen-presentation loss, and FAK-mediated immune exclusion, supported by harmonized multi-omics datasets and batch-aware analytical pipelines. Together, these efforts will be essential for translating TIME biology into biomarker-guided precision therapy for melanoma.

## Figures and Tables

**Table 1:** Summary of resistance mechanisms, TIME effects, biomarkers, evidence levels, and therapeutic implications in BRAFi/MEKi-resistant melanoma.

Resistance Mechanism/Stage	Effect on TIME	Related Biomarkers	Evidence Level and References	Therapeutic Implications
**Early on-treatment immune-permissive window**	Melanoma antigen expression↑, CD8^+^ T-cell infiltration↑, IL-6/IL-8↓; concurrent induction of PD-L1, PD-1, TIM-3	CD8, PD-L1, PD-1, TIM-3, IL-6, IL-8	Clinical evidence from paired biopsies [16,21,23]	Supports early ICI addition or sequential/sandwich strategies
**MAPK reactivation and bypass signaling (NRAS, BRAF amplification, MEK mutations, EGFR/IGF1R/AKT-mTOR, STAT3/Src)**	Restores tumor survival and reshapes cytokine/chemokine output, promoting immune escape	NRAS/BRAF/MEK alterations, RTKs, STAT3, Src signatures	Preclinical + translational evidence [11,24,25,26,28,29,34]	Suggests biomarker-driven combination therapy and adaptive trial design
**Antigen presentation/MHC-I downregulation**	Antigen visibility↓, CD8^+^ T-cell infiltration↓, association with ICI resistance	MHC-I, antigen-presentation genes, CD8	Clinical correlative + pathology evidence [36,39]	Restoration of antigen presentation or use of non-MHC-I-dependent immune strategies
**Dendritic cell dysfunction/GLI1-CCL7 axis**	Impaired DC infiltration, maturation, and T-cell priming; context-dependent immune modulation by MAPK inhibition	DC maturation markers, CCL7, co-stimulatory molecules	Mostly preclinical evidence [24,42]	Timing-sensitive targeted-ICI combinations; DC-supportive approaches
**Cytokine/myeloid remodeling and checkpoint induction**	M2 TAMs, MDSCs, Tregs, checkpoint upregulation, T-cell dysfunction	IL-8, IL-1β, MDK, Tregs, TAMs, MDSCs, TIM-3	Mixed clinical + preclinical evidence [16,25,26,43]	ICI combinations plus myeloid/cytokine-targeting strategies
**FAK/ECM-mediated immune exclusion**	Physical and functional immune barrier, invasion/migration phenotype, resistance to targeted and immune therapy	FAK, RhoA-FAK-AKT, focal adhesion signature, ECM genes	Preclinical/transcriptomic evidence [38,45]	FAK inhibition plus RAF-MEK clamp therapy
**Lymphatic compartment/perilymphatic adipose tissue**	Altered antigen drainage, immune-cell trafficking, and suppressive stromal-adipose crosstalk	LYVE1, PROX1, VEGF-C/FLT4, CCL21, TDLN immune signatures	Clinical correlative + preclinical evidence [46,47,48,49]	Spatially informed biomarker development; therapies preserving/restoring functional lymphatic drainage
**Metastatic-site heterogeneity (brain vs. liver vs. lung/LN)**	Immune infiltration, checkpoint expression, and spatial distribution vary depending on the metastatic organ	CD3, PD-1, TIM-3, PD-L1, TIL density, spatial proximity	Clinical correlative + spatial translational [26,60,61]	Biomarker/site-aware stratification; brain/liver should be analyzed separately
**Longitudinal monitoring biomarkers**	Captures evolving TIME states across treatment course	ctDNA/BRAF VAF/nVAF, LDH, dermoscopic regression, MelanoDB, ireRNAs	Clinical + computational evidence [8,9,62,65,68,69,70]	Baseline/on-treatment/progression sampling to guide sequencing or switching

**Table 2:** Metastatic site heterogeneity in melanoma.

Site	Immune Phenotype	Biomarker Implication	Therapeutic Implication
Liver	Low T-cell infiltration, higher TIM-3	TIM-3, TIL, PD-1 context-dependent	Consider intensified combination/TIM-3-oriented strategies [26]
Brain	Low T-cell and macrophage infiltration; BBB/central nervous system (CNS) constraints	PD-1, CD103/TIL interpret with caution	Prioritize CNS-aware sequencing and immunotherapy integration [72,73,74]
Lung/lymph node	More inflamed/higher immune infiltration	Biomarkers may perform better here	More favorable response context [60]

**Table 3:** Time-resolved TIME remodeling under BRAFi/MEKi in melanoma.

Timing	Main Phenotype of TIME	Mechanisms/Pathways	Suggested Tissue/Histological Indicators	Operable Biomarkers	Therapeutic Implications
**Baseline**	Low CD8, immunosuppressive tendency	Intratumoral heterogeneity, baseline ecological niche	CD8, PD-L1, MHC-I	LDH, ctDNA, etc.	Determines the starting point of treatment strategy
**Early on-treatment**	Antigen↑, CD8↑, IL-6/8↓ but PD-L1↑, exhaustion↑ (mixed phenotype)	Adaptive immune resistance	CD8, PD-1/TIM-3, PD-L1	ctDNA dynamics	Window for early combination/intensification of ICIs
**Progression**	Antigen presentation↓, MHC-I↓, CD8↓, Treg/MDSC/TAM↑, stromal barrier	Defects in antigen presentation, myeloid remodeling, FAK/adhesion pathways	Combined evaluation of MHC-I + CD8	Stratification in advanced stage	Requires mechanism-based stratified combinations (e.g., FAK + RAF-MEK clamp, etc.)

## Data Availability

No new data were created or analyzed in this study. Data sharing is not applicable to this article.

## Abbreviations

The following abbreviations are used in this manuscript:

BBB	Blood–brain barrier
BRAFi/MEKi	BRAF and MEK inhibitors
CNS	Central nervous system
CyTOF-IMC	Imaging mass cytometry
DC	Dendritic cell
ECM	Extracellular matrix
EMT	Epithelial–mesenchymal transition
FAK	Focal adhesion kinase
ICIs	Immune checkpoint inhibitors
ILC2	Group 2 innate lymphoid cell
ireRNAs	Immune-related long non-coding RNAs
LDH	Lactate dehydrogenase
MAPKi	MAPK inhibitor
MDK	Midline protein
MDSCs	Myeloid-derived suppressor cells
MHC-I	Major histocompatibility complex class I molecules
nVAF	Normalized VAF
RTKs	Receptor tyrosine kinases
TAMs	Tumor-associated macrophages
TIME	Tumor immune microenvironment
TME	Tumor microenvironment
TRAEs	Treatment-related adverse events
Tregs	Regulatory T cells
VAFs	Variant allele frequencies
